# Changes in Optical Parameters of SiO_2_:TiO_2_ Films Obtained by Sol-Gel Method Observed as a Result of Thermal Treatment

**DOI:** 10.3390/ma14092290

**Published:** 2021-04-28

**Authors:** Jacek Nizioł, Ewa Gondek, Paweł Karasiński

**Affiliations:** 1 Faculty of Physics and Applied Computer Science, AGH University of Science and Technology, al. Mickiewicza 30, 30-059 Krakow, Poland; 2 Department of Physics, Cracow University of Technology, ul. Podchorążych 1, 30-084 Kraków, Poland; egondek@pk.edu.pl; 3 Department of Optoelectronics, Silesian University of Technology, ul. B. Krzywoustego 2, 44-100 Gliwice, Poland

**Keywords:** slab waveguide, sol-gel method, silica-titania film, spectroscopic ellipsometry, refractive index, thin film

## Abstract

The research focused on materials having potential applications in technology of planar evanescent wave sensors. Four samples of binary SiO_2_:TiO_2_ thin films having different titania content were manufactured through the sol-gel method and dip-coating technique on polished silicon substrates. The samples were subjected to repeated heating/cooling protocols. Simultaneously, their optical parameters were monitored by spectroscopic ellipsometry as they evolved under varying temperature. Subsequent analysis confirmed linear dependence of refractive index on titania content, at least in vis-NIR wavelengths, as well as a low value of the thermal expansion coefficient. It was shown that the thickness of SiO_2_:TiO_2_ films decreased as a result of annealing processes, which may be a consequence of reduced porosity.

## 1. Introduction

Integrated optics has been developing since the 1960s [1]. At present, its main areas of application are optical telecommunication [2] and planar evanescent wave sensors [3]. Integrated optics systems for telecommunications applications are designed to operate in the C-band (1530–1565 nm). They are fabricated from silicon (Si) and indium phosphide (InP) [2,4,5]. Silicon and indium phosphide have a high refractive index, which allows them to achieve large-scale integration of produced systems. The TriPlex platform based on silica nitrate (Si_3_N_4_) [6], capable of operation in the vis-NIR spectral range, is a complement to the two previously mentioned materials platforms. Planar integrated circuits applied in the design of evanescent wave sensors are mainly operating in the vis spectral range [3]. Waveguide films designed to be a part of planar evanescent wave sensors should not only have high refractive index [3,7] but should also possess chemical resistance [8]. SiO_2_:TiO_2_ waveguide films (alternatively referred to as silica-titania) that were developed at the Silesian University of Technology and that fulfil the requirements listed above are the subject of this paper. Moreover, these films have extremely low optical losses and show stability of parameters over long time periods [9]. Their usefulness was proved by showing that the manufacturing of grating couplers, rib waveguides, and directional couplers is possible by using them [7,10].

Residual porosity is an inherent feature of waveguide films fabricated by using the sol-gel method, as well as by other methods of which the physical vapor deposition (PVD) and chemical vapor deposition (CVD) are to be mentioned. The waveguide sensitivity is subject to changes in ambient conditions, e.g., changes in humidity. The influence of humidity on refractive index of waveguide films was observed by Lukosz [11]. The phenomenon of influence of ambient conditions on parameters and properties of planar waveguide films can be critical for operation of resonant structures, e.g., ring resonators or arrayed waveguide gratings (AWG), compliant with a design. That is precisely why it is so useful to know how ambient temperature and humidity affect the material properties of the waveguide film. The need to gain an understanding of how environmental conditions affect the properties of the waveguide films we produce motivated us to undertake the research presented here. The objective of the research presented in this paper was to determine the influence of a cyclic annealing process on the thickness and refractive index of silica-titania (SiO_2_:TiO_2_) films having different content of TiO_2_. Four films having different values of the refractive index, depending on TiO_2_ content, were investigated. The films were fabricated on silicon substrates. Based on our previous investigations we expected that absorption of water would influence the parameters of the investigated films.

Direct methods routinely used to study hydration, such as sorption isotherm [12] or 1H NMR [13], are useless when thin layers are the subject of study because of insufficient sample volume (and mass). This limitation does not apply to ellipsometry, and this technique was used in the reported research to investigate phenomena occurring in SiO_2_:TiO_2_ as a result of thermal treatment.

The paper is organized as follows. In Section 2, we present the procedure for the preparation of samples, the spectroscopic ellipsometry method, and the methodology applied to determine the influence of temperature on samples’ properties. Results of our investigations and their discussion are presented in Section 3, where the influence of temperature on relations between ellipsometric angles as well as on the thickness and refractive index of investigated films is demonstrated. It was shown that the thickness of SiO_2_:TiO_2_ binary films decreased as a result of annealing processes, which may be indirect evidence of reduced porosity.

## 2. Materials and Methods

The sol-gel method is a convenient chemical method allowing synthesis of various materials from liquid solutions of so-called sols. In this study, tetraethoxysilane Si(OC_2_H_5_)_4_ and titanium(IV)ethoxide Ti(OC_2_H_5_)_4,_ referred to as TEOS and TET, were used as precursors to SiO_2_ and TiO_2_, respectively. The hydrolysis and condensation reactions were catalyzed with hydrochloric acid HCl. Ethanol (C_2_H_5_OH) was used as the homogenizing agent. In order to obtain SiO_2_:TiO_2_ films having refractive index values from the middle of the range limited by values typical for SiO_2_ and TiO_2_, the following percentage values for the TiO_2_ content were selected: 50%, 40%, 20%, and 10%. Later in this article, samples will be addressed by these numbers. Precursors were purchased from Sigma-Aldrich, and the remaining reagents were purchased from Avantor Performance Materials, Poland. The prepared sols were deposited on precleaned silicon substrates by using a dip-coating technique. Those substrates were purchased from the Institute of Electronic Materials Technology, Poland. Substrates of this type have well-established parameters; hence, they were chosen to provide the strong refraction index contrast necessary for efficient ellipsometric measurements. The SiO_2_:TiO_2_ layers were air dried and subsequently annealed at a temperature of 500 °C for 60 min. Further details concerning this process can be found elsewhere [9,14]. As a result, visually clear, crack-free, and mechanically stable optical layers were obtained. 

Spectroscopic ellipsometry was used as a convenient tool for investigating the optical properties of the fabricated SiO_2_:TiO_2_ layers. Upon light reflection on a sample, *p*- and *s*-polarizations experience different changes in amplitude and phase. The direct information provided by ellipsometry are two angles $\left(\psi ,\Delta \right)$ defined from the ratio of the amplitude reflection coefficients for *p*- and *s*-polarizations, respectively. Direct inversion of ψ and Δ into refractive index n and extinction coefficient κ is only possible in very few cases. Unknown optical constants and sample thickness can be determined using an iterative numerical procedure. In actual experimental practice, especially when the sample thickness is unknown, the result of such calculations is subject to large uncertainties. The reliability and accuracy of such calculations increase if they include a theoretical model describing dispersion relations of optical constants. Therefore, measurements of ellipsometric angles are carried out for multiple wavelengths and different incident angles [15]. The investigations were carried out using a Woollam M-2000 spectroscopic ellipsometer (J.A. Woollam Co., Lincoln, USA), capable of simultaneously collecting signal at 709 different wavelengths between 1688 nm and 192 nm (0.734 eV to 6.45 eV in energy units). Additionally, two accessories were used. The scanning table allowed position of the sample in XY plane to be changed and guided the probing beam at different angles of incidence. The light spot on the sample surface formed an ellipse of 2 mm by 2–3 mm (depending of the angle of incidence). The size of the spot defined the spatial resolution at which the samples were scanned in the horizontal plane to find a homogenous area in terms of practically invariant ψ and Δ. The second accessory used was a heating table fixed in the thermal chamber. Inside the latter, measurements were only possible at an incidence angle of 70°. The internal temperature was PID controlled, but cooling was achieved by flushing with atmospheric air (or argon), so the control of its rate was limited, especially near ambient temperature. Prior to the experiment, already annealed samples were stored for an “infinitely” long time in laboratory ambient conditions (T = 25 °C, RH = 50%, approximately). 

**Figure 1:**
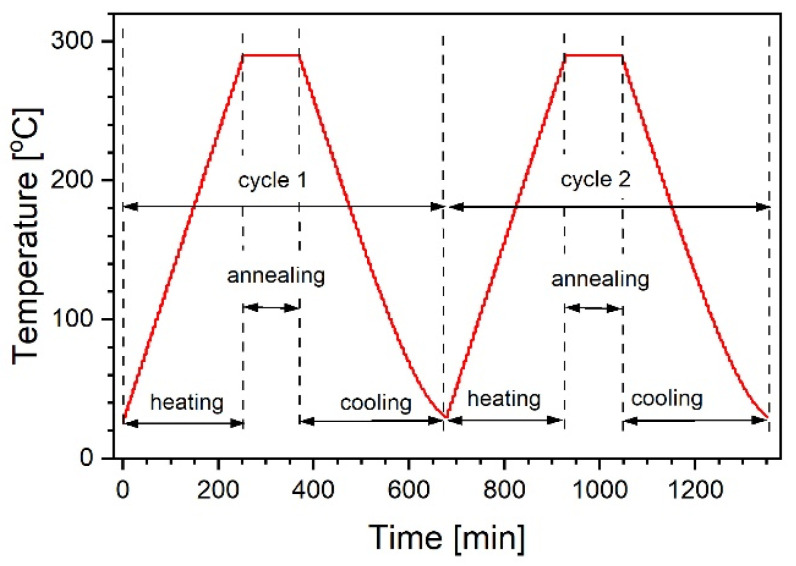
Scheme of the applied heating/cooling protocol. The red line represents temperature variations. The protocol consisted of two identical cycles. At the beginning of the cycle, the temperature was raised in increments of 5 °C and stabilized for 5 min. After reaching 290 °C, annealing continued for 2 h. The cycle ended with cooling, performed in a manner exactly opposite to the heating course. The cycle started and ended at 30 °C. These successive protocol steps are referred to in the subsequent text as first heating, first annealing, first cooling, and the same for the cycle 2.

Four selected samples containing various amounts of TiO_2_ were tested. Each was subjected to a heating/cooling procedure, briefly outlined in Figure 1. During this process, they were continuously studied by spectroscopic ellipsometry and changes in ellipsometric angles as a function of temperature were recorded. To verify temporal variation of the observed effects, the study was repeated after 2 and 30 days for the 20% sample. Meanwhile, the sample was stored under normal laboratory conditions. Evaluation of the preliminary results suggested that the sample released water during the thermal treatment it was subjected to, then reached equilibrium with atmospheric moisture over the next 30 days. To check the effect of atmosphere on the kinetics of water release during the thermal process, the experiment was repeated again in an inert dry argon atmosphere after another 60 days. The applied protocol consisted of only 1 cycle because of constraints related to the equipment. The dispersion of optical functions as a function of temperature was measured for the uncovered substrate to obtain reference data necessary for the analysis of SiO_2_:TiO_2_ layers.

For the qualitative analysis of data provided by spectroscopic ellipsometry, it is necessary to adopt a dielectric function model. Many such models are known from literature [15]. The appropriate choice is usually made empirically, taking into account results obtained previously for similar materials. The chosen model should be as simple as possible and should provide a fit with a low standard deviation. The latter criterion means that adding another free parameter does not significantly improve the fit quality. The Sellmeier model was employed [15] to calculate dispersion of refractive index in the wavelength range where the studied oxides were transparent. Although empirical, this model well characterizes the optical properties of transparent dielectrics, taking into account UV and IR absorption outside the optical window. The Tauc–Lorentz oscillator model was then applied to express the dielectric polarization in the full available NIR/vis/UV spectrum, including absorption bands. The imaginary part of the dielectric permittivity $\varepsilon_{2}$ was modelled from the product of a unique bandgap of amorphous materials [16] and the Lorentz model. The result is referred to as Tauc–Lorentz model in the literature. To further match the real part of the dielectric permittivity $\varepsilon_{1}$, outside of the experimentally available wavelength range (i.e., in IR and vacuum UV), poles (i.e., Lorentz oscillators with zero broadening) were added to the model. Being Kramers–Kronig consistent, such a model was reported to be successfully applied to various amorphous materials [17,18]. 

The sample thickness introduced in the Tauc–Lorentz fits were numerically constrained to be coherent with previously obtained refractive index dispersion and thickness through the Sellmeier model. The goodness of fit was assessed by root mean squared error (MSE) between measured data and model-generated data, summed over all measurement wavelengths. Only such modifications that radically reduced the MSE were considered to further improve the model. The introduction of additional bands is justified if there are reasons other than just improving fit quality [19]. It was not the case for SiO_2_:TiO_2_. 

Numerical analysis of the data in our studies was done using proprietary Wollam software CompleteEase v.6.59 and Origin data processing package v.2020, OriginLab.

## 3. Results and Discussion

**Figure 2:**
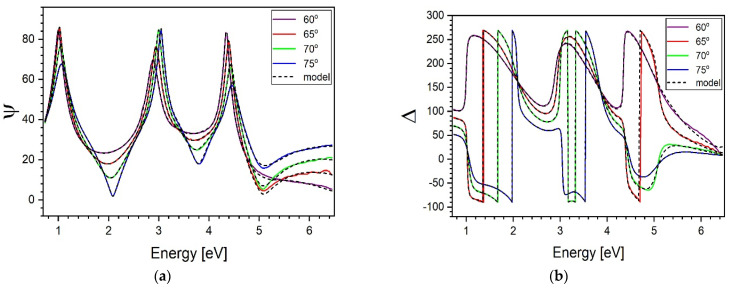
The 20% sample. Ellipsometry angles ψ and Δ (full lines, plots (**a**) and (**b**), respectively) measured at different incidence angles as a function of photon energy. Dashed lines represent curves modelled using Tauc-Lorentz approximation (discussed in the further text).

An example of a typically recorded dispersion of ellipsometric angles ψ and Δ at different incidence angels is shown in Figure 2.

**Figure 3:**
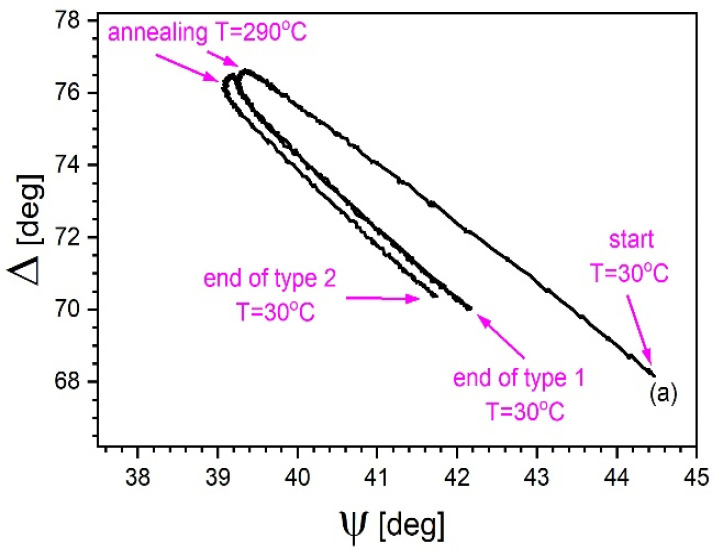
The 20% sample. Dependence of Δ over ψ during the first heating/cooling cycles. The temporal order of presented experimental data is the following: start at 30 °C, heating to 290 °C and annealing, cooling to 30 °C, next heating to 290 °C and annealing, final cooling to 30 °C (see Figure 1). Further in the text, segments of the trace are referred to as “first heating arm”, “first annealing”, “first cooling arm”, “second heating arm”, “second annealing”, and “second cooling arm”, accordingly. Data recorded for 1550 nm, i.e., “fiber optic window” wavelength.

The observed dependence of Δ on ψ, changing as a result of the heating/cooling cycle for the 20% sample is shown in Figure 3. At the end of cycle at room temperature, the measured Δ and ψ do not return to their initial values. The angle Δ plotted as a function of angle ψ for all successive temperatures consists of two horseshoe-shaped curves. The arms of the curve corresponding to cycle 2 are much closer each to the other. The arm representing the second heating basically follows the trace of the preceding first cooling, as if they were mirror images of the same process. It should be noted, however, that they took place practically one after the other. Nevertheless, the arm of the second cooling is slightly shifted. This indicates the role played by annealing at constant temperature of 290 °C, the stage that separates heating from cooling. Virtually identical curves were observed for the other samples. Therefore, they are not discussed here separately.

**Figure 4:**
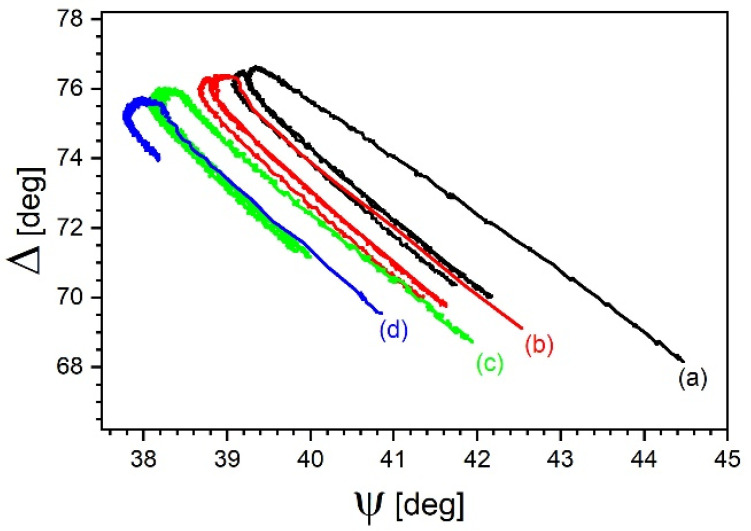
Extension of Figure 2 for the 20% sample. Dependence of Δ over ψ during consecutive heating/cooling protocols—(a) the first one, (b) after two days, (c) after one month, (d) after next 2 months (in argon atmosphere). Data for wavelength of 1550 nm.

A natural continuation was to verify if the observed behavior was reproducible. Experiments rerun for the 20% sample at time intervals described in Section 2, provided the data plotted in Figure 4. As can be seen, two-horseshoe patterns moved in a regular manner as a result of repeated experiments. The distance separating first heating and first cooling arms is always wider than in cycle 2. In each trace, the second heating arm is practically superposed on the first cooling one. The first heating arm in each trace is always longer and slightly displaced with respect to the second cooling arm of the previous trace. If nothing happened between the two experiments, one would expect them to be identical. Since this was not the case, it means that this was the result of processes occurring during storage.

One of the potentially responsible factors was atmospheric moisture. It was eliminated in the experiment carried out in dry argon instead of in atmospheric air, which substantially changed the observed behavior. Indeed, in this case the “cooling” arm was proportionally much shorter than the “heating’ arm, as shown in trace (d) in Figure 4. 

Although the latter is not direct evidence, it makes the assumption more convincing. Both SiO_2_ and TiO_2_ are capable of forming hydrogen bonds, so the occurring process can be imagined as follows. Water molecules become trapped on the surface of the sample already during cooling and form a layer of strongly bound water, which alters the optical properties the sample. For this reason, the cooling arm was shorter in the experiment conducted in dry argon. Prolonged exposure to atmospheric moisture results in a slower buildup of secondary, less strongly bound water layers. This in turn may account for the different values of the (Δ, ψ) pairs measured with a time delay separating the end of one experiment and the start of the next one. Such a process of formation of water layers is known to occur in many hydrophilic systems, especially organic ones [12,13].

**Figure 5:**
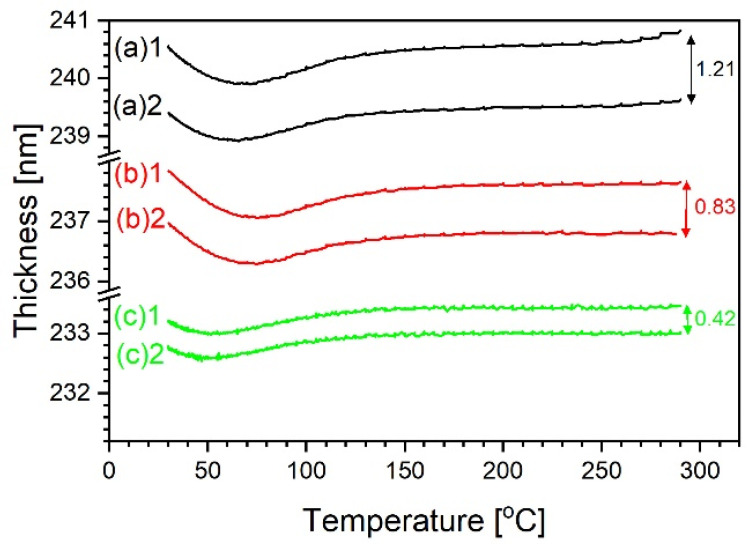
The 20% sample. Thickness variation as function of decreasing temperature, calculated by fitting Sellmeier model to data recorded during first and second coolings (letters refer to successively repeated measurements as in Figure 4, the number stands for the first or the second cooling).

**Figure 6:**
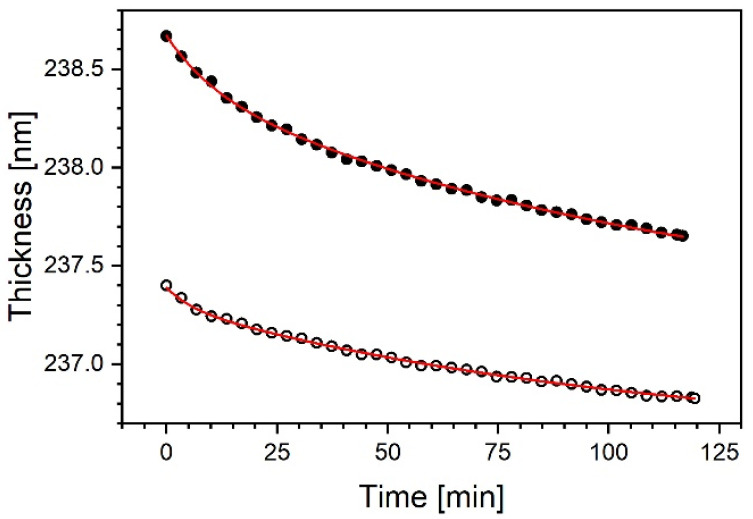
The 20% Sample. Thickness variation as function time at 290 °C, calculated for first (full circles) and second annealing (open circles) using Sellmeier model for data (b) in Figure 4. Continuous lines are fits of a double exponential decay.

Taking into account this hypothesis, if one wants to analyze variation of sample thickness as function of temperature, it is better to compare results obtained during the cooling. The sample, being annealed at 290 °C, is devoid of water, which can be reabsorbed no sooner than well below its boiling point. Figure 5 shows such graphs for the 20% sample. The distance between the thickness at the beginning of first and second cooling within the same experiment decreases, as if the mechanism responsible were losing intensity. Therefore, it can be reasonably assumed that the thickness is lost principally during the annealing process. In order to find this out, it is convenient to represent the relevant data from Figure 4 as a function of time, as in Figure 6.

An example of such data, obtained for the 20% sample during two successive annealing phases (curve (b) in Figure 4), is shown in Figure 6. The double exponential decay function (Equation (1)), in a satisfactory manner illustrates the decrease in the sample thickness (*d*) as a function of time (*t*). The results are set out in Table 1 and suggest the presence of two processes. The second time constant is about 10 times longer than the first one in both annealings. A similar picture can be derived from the relevant data of the other experiments in the series. The value of the second time constant can be used to calculate the minimum length of annealing to obtain a stable thickness.

(1) $$d=A_{1}\times exp\left(-\frac{t}{\tau_{1}}\right)+A_{2}\times exp\left(-\frac{t}{\tau_{2}}\right)+d_{0}$$

**Figure 7:**
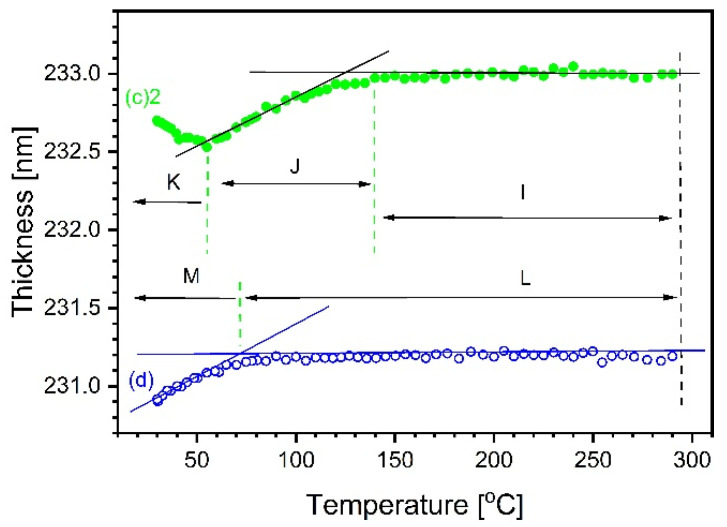
The 20% sample. Thickness variation as function of decreasing temperature in atmospheric air—full, green circles as (c)2 in Figure 5, and in argon—open, blue circles (from data (d) in Figure 4). Results of fitting Sellmeier model to data measured in wavelength range between 1688 nm and 350 nm.

To investigate the role of atmosphere, in Figure 7 are compared variations of thickness observed during the cooling in air to those in argon (outcome of the next experiment). Both curves consist of practically flat fragments at temperatures superior to 100–120 °C (I and L), followed by linear inclined fragments (J and M). It is difficult to convincingly explain the reason for the flat fragments. 

The sloped fragment corresponds to a typical thermal expansion. Its coefficient is of the order of about $5–7\times 10^{-6}$ °C^−1^, which is a rather low value. In contrast to (d), the thickness in c(2) starts to climb again at temperatures inferior to 60–70 °C. Such a rise of a few tenths of a nanometer can come from water continuously accumulating on the sample surface around hydrophilic sites. It is impossible to numerically separate such a thin layer, 3 orders of magnitude thinner than the oxide layer, in the model of optical constants. It is therefore only a plausible hypothesis; however, it is supported by similar observations for analogous systems. For example, the combination of TEOS and a less polar compound, 1,2-bis(triethoxysilyl)ethane, removed this effect [20]. The alternative hypothesis assuming a negative expansion coefficient for TiO_2_ below 50 ℃ seems not plausible.

**Figure 8:**
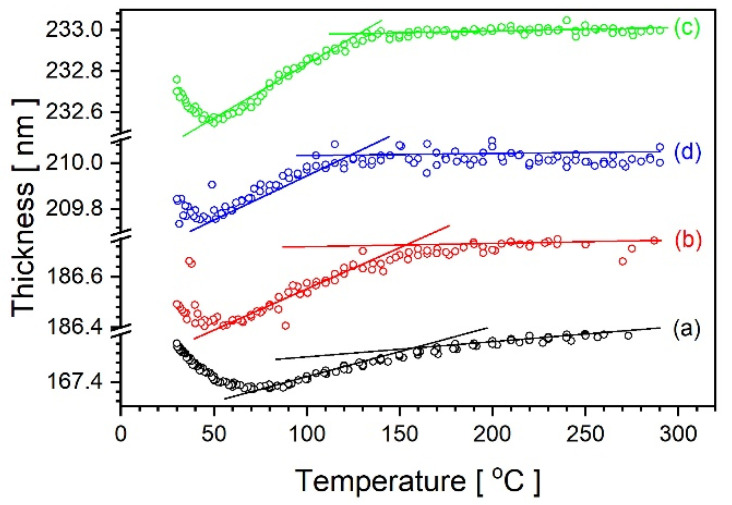
Thickness variation as a function of decreasing temperature for samples 50%—(a), 40%—(b), 20%—(c), 10%—(d) calculated using Sellmeier model in wavelength range between 1688 nm and 350 nm and data measured after completion of heating/cooling procedures.

Roughly similar variation in thickness was observed for the other samples, as illustrated in Figure 8. Minor differences appear to be justified by the previous history of the sample.

**Figure 9:**
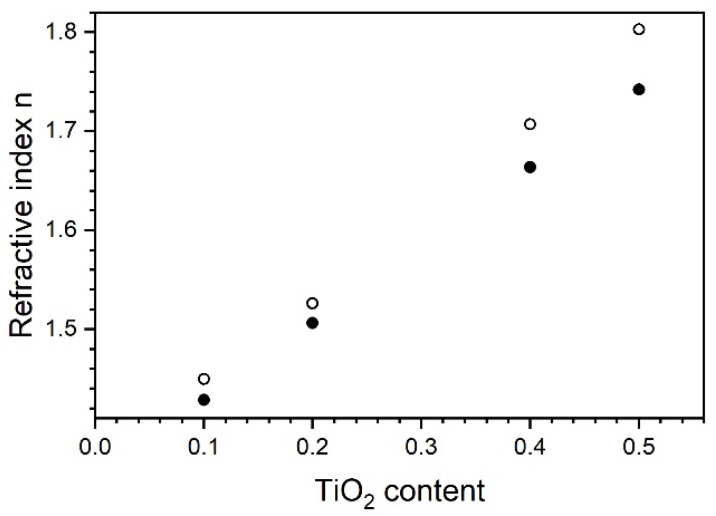
Dependence of refractive index on TiO_2_ content. It was calculated using Sellmeier model in wavelength range between 1688 nm and 350 nm and data measured after completion of heating/cooling procedures. The diagram shows chosen examples—full circles for 1550 nm (C-band), open circles for 589 nm (sodium doublet). A similar dependence occurred for other wavelengths.

Given the above fact, the refractive index dispersion in the transparent window (1688 nm to 350 nm, i.e., up to 3.5 eV) was calculated within the Sellemeier model framework for data recorded at temperature of 50 °C, after the completion of heating/cooling protocol (containing a total of 4 h annealing at 290 °C). The found values were proportional to titanium dioxide content. Figure 9 shows a plot of refractive indices in which example wavelengths are plotted versus content of TiO_2_ in the sample. Points are arranged practically along a straight line. If this line were extrapolated at TiO_2_ content equal to 0% or 100%, one would get an estimate for refractive indices of either pure SiO_2_ or TiO_2_. These results are confirmed in the literature, assembled in Table 2. The estimates fall inside this compilation, which confirms the validity of the assumption. 

In Figure 2 are shown ψ and Δ typically measured for the 20% sample using a scanning table and at different angles of incidence. A minimal, hardly visible discrepancy between experimental and fitted Tauc–Lorentz curves exists at energies superior to ca. 5 eV, equivalent to 250 nm. This fact in not surprising. As can be observed in Figure 2, in this spectral region the occurring absorption significantly reduces the intensity of the light reflected from the lower surface (adjacent to the substrate) of the studied oxide layers. However, it does not vanish completely. Numerical modelling carried out as if the light were reflected from the upper surface leads to unphysical results. Consequently, data recorded near the vacuum UV suffer the strongest uncertainty. 

**Figure 10:**
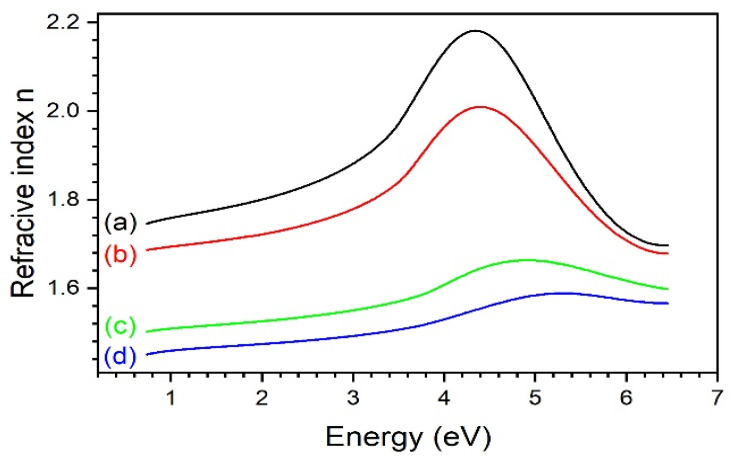
Refractive indices derived within the framework of Tauc–Lorentz model for samples 50%—(a), 40%—(b), 20%—(c), 10%—(d) at 50 °C.

The Tauc–Lorentz model, averaged for several adjacent points on the sample, was further iteratively fitted to data measured during the heating/cooling protocol. Some values of the fitted parameters are compared in Table 3. One can see that decreasing content of TiO_2_ moves the center energy of the band toward higher energies and simultaneously widens the bandgap. For lower TiO_2_ content (10% and 20%), temperature causes an increase in band energy, while for higher content (40% and 50%) the opposite effect is observed. Dispersion of refractive index deduced from Tauc–Lorentz modelling are shown in Figure 10.

As mentioned in Section 1, SiO_2_:TiO_2_ films prepared using the sol-gel technique contain voids. In waveguides they are a parasitic feature, a potential source of light dispersion. Their content is always a subject of speculation because none of the available techniques are able to measure the parameter directly. Thermal evolution of optical parameters can be used as a convenient tool to access in real time the collapsing of caverns in such films. For the 20% sample, two sets of dispersions representing $\left(n,\kappa \right)$ were calculated for the experimental data, measured at the first and last moment the sample was annealed at 290 °C (i.e., after a total of 14 h). The second one was considered as a continuous material and the first one as a material with voids. Assuming no chemical changes occurred at temperatures of 290 °C and using effective medium, the approximation of the difference in voids content was estimated as 3.3%.

## 4. Conclusions

The reported research revealed a linear dependence of the refractive index on the composition of SiO_2_:TiO_2_ layers obtained by the sol-gel technique, at least for a wavelength range between 1688 nm and 350 nm. Regardless of TiO_2_ content, thermal expansion of the investigated materials was low, which confirms their leading position among materials of potential interest for integrated optics. Such a property is an asset in tailoring materials for planar waveguides.

It was demonstrated through indirect evidence that moisture accumulation on the SiO_2_:TiO_2_ layers in contact with atmospheric air did not occur in dry inert gas (argon). Spectroscopic ellipsometry was proposed for use as an effective tool for estimating the content of voids. Voids in waveguides are parasitic features, a potential source of light dispersion. Although this drawback of sol-gel processed layers had been known, it was difficult to assess in real time the rate of voids removal by heat treatment. In a practical application there always exists an imperative to choose between quality and economy. The proposed method will make this choice less frustrating.

## Figures and Tables

**Table 1:** Double exponential decay parameters, fitted to the data in Figure 5.

Parameter	First Annealing	Second Annealing
$d_{0}$ [nm]	237.22 ± 0.05	236.60 ± 0.02
$A_{1}$ [nm]	0.253 ± 0.018	0.094 ± 0.007
$\tau_{1}$ [min]	10.9 ± 1.0	5.7 ± 0.8
$A_{2}$ [nm]	1.19 ± 0.03	0.70 ± 0.02
$\tau_{2}$ [min]	113 ± 10	107 ± 6

**Table 2:** Examples from the literature on the refractive index of SiO_2_ and TiO_2_, measured for different types of thin films.

	$n\left(\lambda \;=\;589\;\mathrm{nm}\right)$	$n\left(\lambda \;=\;1550\;\mathrm{nm}\right)$	Remarks
SiO_2_	1.355	1.350	(found by extrapolation in Figure 9)
	1.4584	1.4440	(fused silica) [21]
	1.4733	1.4657	580 nm thin film deposited on BK7 substrate using magnetron sputtering technique [22]
	1.4776	1.4694	300 nm thin film deposited on fused silica using the ion assistance electronic beam deposition technique [23]
	1.4648	1.4574	reactive electron-beam evaporation onto various sorts of substrates [24]
TiO_2_	2.237	2.135	(found by extrapolation in Figure 9)
	2.1460	2.0567	Titanium(IV) oxide (Titanium dioxide, TiO_2_) thin film (thickness 200 nm) on glass substrate [25]
	2.4108	2.2808	film prepared by atomic layer deposition, [26]

**Table 3:** Selected parameters fitted by the Tauc–Lorentz model. The fits were performed using data recorded when the sample was at 290 °C or 50 °C for the last time in the experiment.

Sample	Temperature	Band Center Energy	Bandgap Energy
50%	290 °C	5.01	3.14
	50 °C	4.57	3.46
40%	290 °C	5.13	3.26
	50 °C	4.53	3.56
20%	290 °C	5.018	4.10
	50 °C	5.00	3.82
10%	290 °C	5.21	4.19
	50 °C	5.65	3.86

## Data Availability

The data presented in this study are available on request from the corresponding author.
